# Interactive effects of exercise intensity and recovery posture on postexercise hypotension

**DOI:** 10.1152/ajpregu.00036.2024

**Published:** 2024-04-22

**Authors:** Xueer Lu, Richie P. Goulding, Toby Mündel, Zachary J. Schlader, James D. Cotter, Shunsaku Koga, Naoto Fujii, I-Lin Wang, Ziyang Liu, Hao-Yu Li, Hui Wang, Huixin Zheng, Narihiko Kondo, Chin-Yi Gu, Tze-Huan Lei, Faming Wang

**Affiliations:** ^1^College of Physical Education, Hubei Normal University, Huangshi, People’s Republic of China; ^2^Shenzhen Nanshan Qianhai Era No.2 Kindergarten, Shenzhen, People’s Republic of China; ^3^Department of Human Movement Sciences, Faculty of Behavioral and Human Movement Sciences, Amsterdam Movement Sciences, Vrije Universiteit Amsterdam, Amsterdam, The Netherlands; ^4^Department of Kinesiology, Brock University, St. Catharines, Ontario, Canada; ^5^Department of Kinesiology, Indiana University School of Public Health Bloomington, Bloomington, Indiana, United States; ^6^School of Physical Education, Sport and Exercise Sciences, University of Otago, Dunedin, New Zealand; ^7^Laboratory for Applied Human Physiology, Graduate School of Human Development and Environment, Kobe University, Kobe, Japan; ^8^Faculty of Sport and Sciences, University of Tsukuba, Tsukuba, Japan; ^9^Centre for Translational Research, University of Otago, Wellington, New Zealand; ^10^Centre for Molecular Biosciences and Non-Communicable Diseases, Xi'an University of Science and Technology, Xi'an, China

**Keywords:** postexercise baroreflex, orthostatic stress, postexercise syncope, syncope

## Abstract

Postexercise reduction in blood pressure, termed postexercise hypotension (PEH), is relevant for both acute and chronic health reasons and potentially for peripheral cardiovascular adaptations. We investigated the interactive effects of exercise intensity and recovery postures (seated, supine, and standing) on PEH. Thirteen normotensive men underwent a V̇o_2max_ test on a cycle ergometer and five exhaustive constant load trials to determine critical power (CP) and the gas exchange threshold (GET). Subsequently, work-matched exercise trials were performed at two discrete exercise intensities (10% > CP and 10% < GET), with 1 h of recovery in each of the three postures. For both exercise intensities, standing posture resulted in a more substantial PEH (all *P* < 0.01). For both standing and seated recovery postures, the higher exercise intensity led to larger reductions in systolic [standing: –33 (11) vs. –21 (8) mmHg; seated: –34 (32) vs. –17 (37) mmHg, *P* < 0.01], diastolic [standing: –18 (7) vs. –8 (5) mmHg; seated: –10 (10) vs. –1 (4) mmHg, *P* < 0.01], and mean arterial pressures [–13 (8) vs. –2 (4) mmHg, *P* < 0.01], whereas in the supine recovery posture, the reduction in diastolic [–9 (9) vs. –4 (3) mmHg, *P* = 0.08) and mean arterial pressures [–7 (5) vs. –3 (4) mmHg, *P* = 0.06] was not consistently affected by prior exercise intensity. PEH is more pronounced during recovery from exercise performed above CP versus below GET. However, the effect of exercise intensity on PEH is largely abolished when recovery is performed in the supine posture.

**NEW & NOTEWORTHY** The magnitude of postexercise hypotension is greater following the intensity above the critical power in a standing position.

## Graphical abstract

**Figure:**
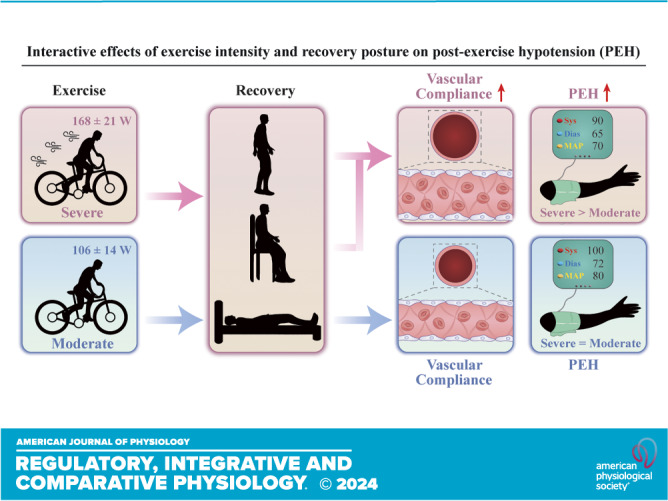

## INTRODUCTION

After dynamic exercise, arterial blood pressure drops below baseline for up to 24 h (1), a phenomenon known as postexercise hypotension (PEH), which is partially attributable to the downward resetting of baroreflex activity (1, 2). Specifically, this phenomenon may be attributed to the combined effects of humorally mediated postexercise skeletal muscle vasodilation and a centrally mediated reduction in sympathetic outflow from the rostral ventrolateral medulla to the sympathetic adrenergic nerves, leading to diminished vasoconstrictor activity (3–5). The occurrence of PEH is considered to be an important exercise outcome for contributing to both the health-related reduction in blood pressure and thereby improvement of cardiovascular health (6), and the exercise-mediated expansion of blood volume (7, 8).

Our previous findings indicated that performing exhaustive constant load exercise above critical power (i.e., severe intensity exercise) resulted in a greater reduction in systolic, diastolic, and mean arterial blood pressures compared with work-matched exercise performed at intensities below critical power (i.e., heavy and moderate) (9). This greater PEH was accompanied by greater increases in vascular compliance (9). This underscores cardiovascular health effects of high-intensity exercise training in normotensive populations. It is important to note, however, that PEH may lead to postexercise syncope if individuals remain motionless in an upright posture after exercise, with approximately 50%–80% of healthy individuals experiencing presyncopal symptoms under such conditions (10).

The combined effects of exercise intensity and orthostasis on PEH may be related to the severity of the orthostatic stress. For example, Scott et al. (11) found that under moderate orthostatic stress, induced by lower body negative pressure (–40 mmHg), higher exercise intensities (120% of maximal oxygen uptake, V̇o_2max_) did not result in a greater reduction in mean arterial pressure (MAP) and cerebral blood flow compared with exercise performed below the gas exchange threshold (GET). However, Mündel et al. (12) utilized a progressive increment of lower body negative pressure until the onset of presyncope, revealing that the time to presyncope was faster after exercising at 70% heart rate reserve compared with 30% heart rate reserve. It is noteworthy that the total work done in the 70% heart rate reserve trial was greater than in the 30% heart rate reserve trial, and both studies used lower body negative pressure tests instead of postural-induced hypotension. Recognizing that lower body negative pressure may not fully replicate the effects of postural hypotension in real-life situations due to differences in the hydrostatic gradient and baroreceptor unloading, the combined effect of exercise intensity and orthostatic stress induced by posture on PEH remains unknown and warrants further investigation (13).

Addressing these issues is crucial, as it could potentially elucidate whether a standing recovery posture following high-intensity exercise poses a greater risk of postexercise syncope and/or greater stimulus for positive cardiovascular effects, compared with moderate-intensity exercise (i.e., below GET). Moreover, the exploration of whether the exercise-dependent impact of PEH can be modified by varying recovery postures remains unexplored. Specifically, adopting standing recovery posture following exercise above the critical power threshold may induce greater gravitational venous pooling, potentially leading to a more pronounced manifestation of PEH. Nevertheless, Guazzi et al. (14) demonstrated greater brachial artery flow-mediated vasodilation in a 60° head-up tilt compared with a supine posture. Considering our previous findings that exercise intensity above critical power resulted in enhanced postexercise vascular compliance compared with intensities below critical power (9), it is possible that the threshold intensity-based postexercise vasodilation is influenced by the combined effects of exercise intensity and orthostatic stress. However, this matter remains unresolved and necessitates further investigation.

The aim of this study was to investigate the combined effect of postures and exercise intensity on the magnitude of PEH in normotensive men. Given our previous findings, which indicated that the magnitude of PEH was greater with exercise above critical power when compared with exercise below the GET (9) and considering that the gravitational pooling is greater during standing than seated and supine recoveries (15), we hypothesized that performing exercise above critical power with a standing recovery posture would result in a greater magnitude of PEH compared with the exercise intensity below GET in the same standing posture. Furthermore, within each exercise intensity, we anticipated that the magnitude of PEH would be greater in standing recovery posture compared with seated and supine recovery postures.

## METHODS

### Participants

An a priori power analysis (G*Power version 3.1.9.4; Heinrich Heine University Düsseldorf, Düsseldorf, Germany) showed that a minimum of eight participants was required based on conventional α (0.05) and β (0.80) values and an effect size of 0.51 as reported by Mündel et al. (12) using the postexercise reduction in MAP as the primary dependent variable. Therefore, 13 healthy, recreationally active but untrained, normotensive (Table 1) Asian men were recruited for this study. Although recruitment was intended to target both males and females, the three females who initially took part in the study dropped out following the critical power determination trials. This study was approved by the Hubei Normal University human ethical committee and registered on the China clinical trial database ChiCTR2300071885 with each participant provided written and informed consent.

**Table 1.** Participants’ characteristics

Participant	Age, yr	Systolic BP, mmHg	Diastolic BP, mmHg	V̇o_2max_, L·min^–1^	Power Output, W
1	25	126.5	80	3.90	338
2	25	119	79	3.30	300
3	25	125	77	2.50	255
4	23	138	81	3.55	245
5	24	135	79	3.0	275
6	24	115	79	3.50	279
7	24	110	77	3.14	254
8	24	130	86	2.60	213
9	22	125.5	76	3.30	295
10	24	116	82	3.70	286
11	24	134.5	84.5	2.20	215
12	24	114	79	2.13	227
13	37	120	89	2.85	265
Mean	25	124	81	3.0	265
SD	3.7	8.9	3.8	0.60	35.9

BP, blood pressure.

## EXPERIMENTAL OVERVIEW

All participants reported to a temperature-controlled laboratory (24.5 ± 0.5°C, 50 ± 5% relative humidity) on 12 occasions and performed *1*) a maximal ramp incremental exercise test, *2*) five critical power determination trials, and *3*) six experimental trials comprising a full crossover of two intensities and three recovery postures, with arterial blood pressure measured during exercise and recovery. The two exercise intensities were 10% above critical power (i.e., 10% > CP; severe) and 10% below the GET (i.e., 10% < GET; moderate). The three recovery postures were seated, supine, and standing. The order of recovery postures was randomized and counterbalanced while participants first performed the 10% > CP trials toward volitional exhaustion and then performed the 10% < GET trials (this sequence was necessary to ensure matched work volume across intensities). Each trial was separated by at least 48 h to ensure adequate recovery. Each experimental trial was conducted at the same time of day to eliminate the effect of diurnal rhythm on blood pressure and body temperature fluctuations (16) and performed 2 h postprandial.

### Ramp Incremental Testing and the Critical Power Trials

All participants completed a maximal ramp incremental exercise test on a cycle ergometer (Ergoline) for determination of V̇o_2max_, the GET (VT1, a noninvasive surrogate for the lactate threshold) and the power outputs for subsequent visits. Each test consisted of a 4-min baseline period of cycling at 20 W, followed by a ramped, linear increase in work rate of 30 W/min at a fixed cadence of 70 rpm until the participant could no longer maintain the cadence above 60 rpm despite strong verbal encouragement. Ventilatory and gas exchange variables were measured continuously. V̇o_2max_ was confirmed by either *1*) the occurrence of a plateau in V̇o_2_ despite an increasing workload during the ramp incremental test or *2*) the occurrence of a plateau in the plot of V̇o_2_ versus power output when determined from the discontinuous tests used to determine critical power. On a separate day, a validation trial was conducted to ensure the accuracy of the V̇o_2max_ measurement with both trials being nonsignificant to each other (all *P* > 0.3). The GET was independently verified by two different investigators (T.-H.L. and R.P.G.) using the following criteria: *1*) a disproportionate increase in CO_2_ production (V̇co_2_) relative to V̇o_2_; *2*) an increase in the ventilatory equivalent for O_2_ (i.e., V̇e/V̇o_2_) without an increase in the ventilatory equivalent for CO_2_ (i.e., V̇e/V̇co_2_); and *3*) an increase in end-tidal O_2_ tension with no change in end-tidal CO_2_ tension.

Approximately 1 wk following the recovery from the maximal ramp incremental exercise test, all participants performed five constant workload trials to determine the power-duration relationship for the determination of the critical power and *W*′ (finite work capacity available above critical power) according to Jones et al. (17). Specifically, the five constant power output trials were selected to ensure a range of times to exhaustion that spanned between 2 and 15 min, and each trial was separated by at least 48 h. These power outputs were initially selected based on performance in the incremental test and calculated to be in the range of 50%Δ (i.e., a work rate calculated to require 50% of the difference between GET and V̇o_2max_) to 110% V̇o_2max_. All tests began with a 4-min period of baseline pedaling at 20 W before an abrupt transition to the desired power output was applied, and participants exercised until task failure. Time to task failure was recorded to the nearest second. If the peak V̇o_2_ attained at the end of any given trial was <95% of that attained in the incremental test, that trial was repeated. Conversely, if the peak V̇o_2_ attained at the end of any given trial was >105% of that attained in the incremental test, the incremental test was repeated. In all cases, V̇o_2max_ was verified.

### PEH Trials with Different Recovery Postures

The two different exercise intensities used in the PEH trials were power outputs selected to elicit physiological responses consistent with moderate and severe intensity exercise. The exercise duration of the 10% < GET trials was modified to ensure that the total work done in each trial was the same as the 10% > CP trial (18). On separate occasions, upon the completion of each exercise intensity trial, participants were told to perform seated, supine, or standing recovery throughout the 1-h recovery period. The standing recovery was performed by asking the participants to stand upright immediately following exercise for 1 h. Participants were told to maintain the standing posture with both of their hands supported by the handlebar of the cycle ergometer. The seated recovery was performed on the cycle ergometer, whereas the supine recovery was performed on a custom-made medical gurney.

## MEASUREMENTS

### Cardiovascular

Heart rate was recorded continuously from the detection of R-R intervals (Polar Vantage XL; Polar Electro, Kempele, Finland), whereas brachial artery blood pressure was measured manually via sphygmomanometry. Blood pressure measurements were performed in duplicate by the same experienced operator at rest, end exercise, and every 5 min during the recovery period. Mean arterial pressure (MAP) was calculated as diastolic blood pressure + 1/3 pulse pressure. Vascular stiffness was estimated via brachial-ankle pulse wave velocity (baPWV, BP-203RPE III; Omron, Japan) before and after 60 min of the recovery as a surrogate of arterial compliance (19). Oxygen pulse was calculated using V̇o_2_/HR.

### Anthropometric

Height and body mass were measured using a stadiometer (Seca, Hamburg, Germany; accurate to 0.1 cm) and scale (Jadever, Taipei, Taiwan; accurate to 10 g).

### Respiratory

Expired respiratory gases were collected from a mixing chamber and analyzed for V̇o_2_, V̇co_2_, and V̇e using online breath-by-breath system (Max II; AEI). Data were recorded breath-by-breath and smoothed using a 10-s average during the ramp incremental test and every 3 s during the subsequent trials. This system was calibrated before each trial using a zero and β-standard gas concentrations and a 3-L syringe (Hans Rudolph 3 L Calibration Syringe), according to the manufacturer’s instructions.

### Body Temperatures

As core temperature (T_core_) can potentially alter postexercise vascular compliance, which could in turn influence the magnitude of PEH (20), we measured T_core_ to provide insight into this potential mechanism. T_core_ was measured by a rectal thermistor (TMQ-DAG; Unimed, China; accurate to 0.1°C). Mean skin temperature (T_sk_) was measured at four sites using calibrated skin thermistors (Grant Instrument, Ltd., Cambridge, UK; accurate to 0.2°C) secured on the calf, thigh, chest, and forearm using surgical tape (3M Healthcare). Regional-weighted mean T_sk_ was calculated according to the equation of Ramanathan (21).

### Hematological Variables

In each session before, immediately after exercise, and following 1 h recovery, capillary blood samples were taken from the fingertip in duplicate. Whole blood was used to measure hemoglobin concentration (Hemo Control; EKF Diagnostics, Germany). Subsequently, hematocrit was calculated and the plasma volume changes were calculated via the Dill and Costill equation (22).

### Data Analysis

Critical power and *W*′ were obtained using the power-time model (*Eq. 1*), linear work-time model (*Eq. 2*), and the linear inverse of time model (*Eq. 3*), where power is plotted against the inverse of time (*Eq. 3*), as follows:

(*1*) $$P=\frac{W^{\prime }}{T}+\text{CP}$$

(*2*) $$W=\text{CP}\times T+W^{\prime }$$

(*3*) $$P=W^{\prime }\times \left(\frac{1}{T}\right)+\text{CP}$$

where *P* is power, *T* is time, *W* is work done, and CP is critical power. The standard error of the parameter estimates for CP and *W*′ were expressed as a coefficient of variation relative to the parameter estimate (CV%). The model with the lowest average CV% was then used for all subsequent analyses.

### Statistical Analysis

As PEH is defined as the postexercise change in MAP relative to the baseline value, systolic (ΔSys) and diastolic blood pressure (ΔDias), and MAP (ΔMAP) were analyzed and presented as the change from baseline value. For clarity, absolute data are also presented. Because of the considerable number of levels for exercise intensity, postures, and recovery time factors, the postexercise minimum values in each of these variables were also analyzed and presented as summary statistics. All statistical analyses were performed with SPSS software for Windows (IBM SPSS Statistics 20), and figures were produced using GraphPad Prism (Prism version 7.00; GraphPad Software). Homogeneity of variance was examined by Levene’s test, and the normality of the data was examined by the Kolmogorov–Smirnov test. Resting systolic, diastolic, and mean arterial pressures during the resting orthostatic challenge test were analyzed by two-way repeated ANOVA, whereas the resting systolic, diastolic, and mean arterial pressures at rest during seated position on the cycle ergometer were analyzed by one-way repeated ANOVA. Similarly, the effect of exercise intensity on the magnitude of PEH, the postexercise minimum values for systolic blood pressure, diastolic blood pressure, and MAP were analyzed by one-way repeated ANOVA. The remaining physiological variables were analyzed using three-way repeated (posture·time·intensities) ANOVA. In all cases, where main or interaction effects occurred, post hoc pairwise analyses were performed using paired *t* test with Bonferroni adjustment where relevant. Data presented are means ± SD, unless stated otherwise. Statistical significance was accepted at *P* < 0.05.

## RESULTS

### Incremental Test and Power-Duration Relationship

V̇o_2max_ was 3.0 ± 0.6 L·min^−1^ (40.5 ± 7.8 mL·kg^−1^·min^−1^), and the GET occurred at 2.0 ± 0.3 L·min^−1^ (24.0 ± 4.9 mL·kg^−1^·min^−1^), with an associated power output of 119 ± 17 W (Table 1). The model producing the best-fit power-duration parameter estimates resulted in a critical power of 153 ± 19 W (1.9 ± 1.1 CV%) and a *W*′ of 18.7 ± 6.7 kJ (9.2 ± 6.2 CV%). Power-duration parameter estimates for each participant, including the predicting model used, are illustrated in Table 2.

**Table 2.** Individual best-fit power-duration relationship parameters

Subject	Best-Fit Model	CP, W	SEE, W	CV, %	*W*′, kJ	SEE, kJ	CV, %
1	Hyperbolic	189	1.6	1	23.0	1.5	7
2	Hyperbolic	157	2.8	2	23.0	1.0	4
3	Hyperbolic	152	2.2	1	13.2	1.3	10
4	1/Time	150	3.2	2	22.9	1.1	5
5	Work time	150	6.4	4	14.3	3.3	23
6	Work time	173	1.2	1	17.6	0.5	3
7	Hyperbolic	144	2.6	2	16.0	1.8	11
8	1/Time	121	4.2	3	15.6	1.2	8
9	Hyperbolic	141	2.8	2	29.8	1.7	6
10	Hyperbolic	157	2.6	2	31.4	1.7	5
11	Hyperbolic	128	1.7	1	10.5	1.3	13
12	Hyperbolic	144	0.9	1	11.9	0.6	5
13	Hyperbolic	181	4.6	3	14.0	2.9	20
	Mean	153	3	2	19	2	9
	SD	19	2	1	7	1	6

CP, critical power; CV, coefficient of variation; SEE, standard error of the estimate.

### Exercise Intensity Estimation

The 10% > CP trial was conducted at 168 ± 21 W. Task failure occurred within 938 ± 200 s (total work done = 158.9 ± 42.8 kJ). V̇o_2peak_ attained in the three 10% > CP trials were not different from each other and were not different from the V̇o_2max_ measured during incremental exercise (*P* = 0.69). The 10% < GET trial was conducted at 106 ± 14 W (duration = 1,504 ± 403 s, total work done = 158.9 ± 42.8 kJ). A steady-state V̇o_2_ indicative of moderate exercise was confirmed in all cases, with no difference in V̇o_2_ between 3 min and the completion of exercise (*P* = 0.14). Furthermore, V̇o_2_ at the end-exercise across the three trials did not differ from each other (*P* = 0.71).

### Postural-Induced Hypotension at Rest

Before exercise, systolic, diastolic, and mean arterial pressures were not different between 10% > CP and 10% < GET (all *P* > 0.40) but were lower in standing posture as compared with seated posture (all *P* < 0.01).

### Effect of Exercise Intensities and Postures on PEH

**Figure 1.**
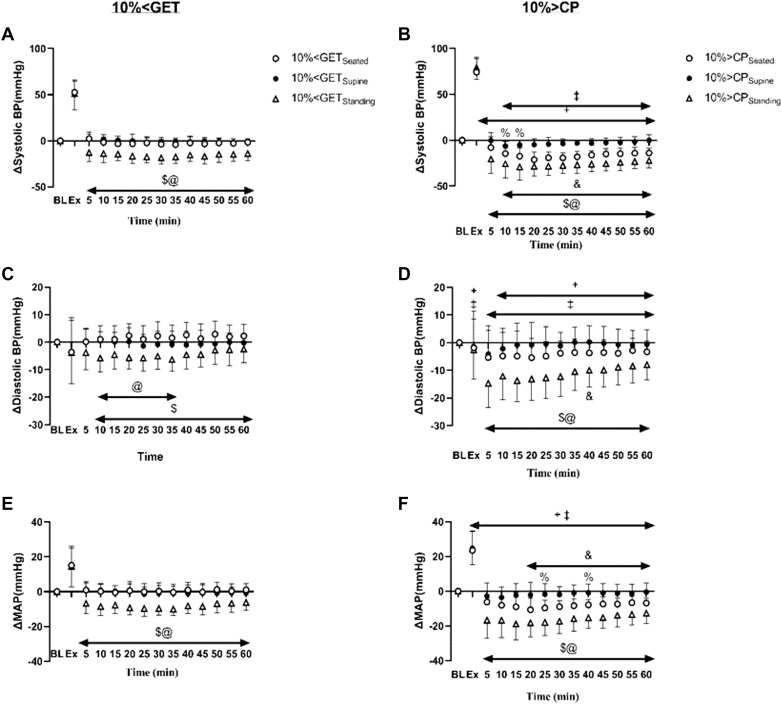
Delta change of systolic, diastolic, and mean arterial pressures from baseline at 10% < GET (*A*, *C*, *E*) and 10% > CP (*B*, *D*, *F*) across different recovery postures (seated, supine, and standing) during exercise and throughout the 1-h recovery. &Significant differences between seated and supine within each intensity (*P* < 0.05); $significant differences between seated and standing within the same exercise intensity (*P* < 0.05); @significant differences between supine and standing within the same exercise intensity; +10% > CP different from 10% < GET at seated position (*P* < 0.05); ‡10% > CP different from 10% < GET at standing position (*P* < 0.05); %significant differences between 10% > CP and 10% < GET at supine position (*P* < 0.05).

**Figure 2.**
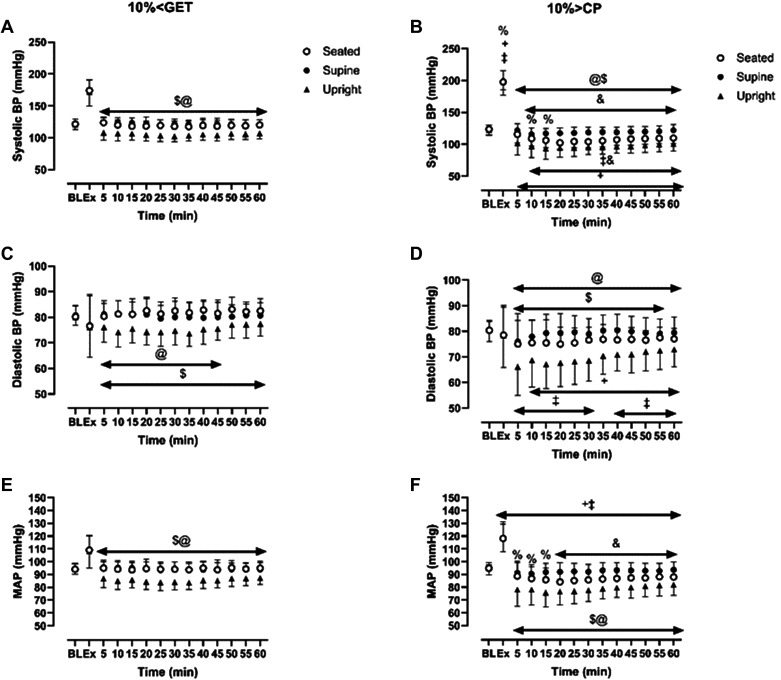
Absolute value of systolic blood pressure, diastolic blood pressure, and mean arterial blood pressure from baseline at 10% < GET (*A*, *C*, *E*) and 10% > CP (*B*, *D*, *F*) across different recovery postures (seated, supine, and standing) during exercise and throughout the 1-h recovery. &Significant differences between seated and supine within each intensity (*P* < 0.05); $significant differences between seated and standing within the same exercise intensity (*P* < 0.05); @significant differences between supine and standing within the same exercise intensity (*P* < 0.05); +10% > CP different from 10% < GET at seated posture (*P* < 0.05); ‡10% > CP different from 10% < GET at standing position (*P* < 0.05); %significant differences between 10% > CP and 10% < GET at supine posture (*P* < 0.05).

**Figure 3.**
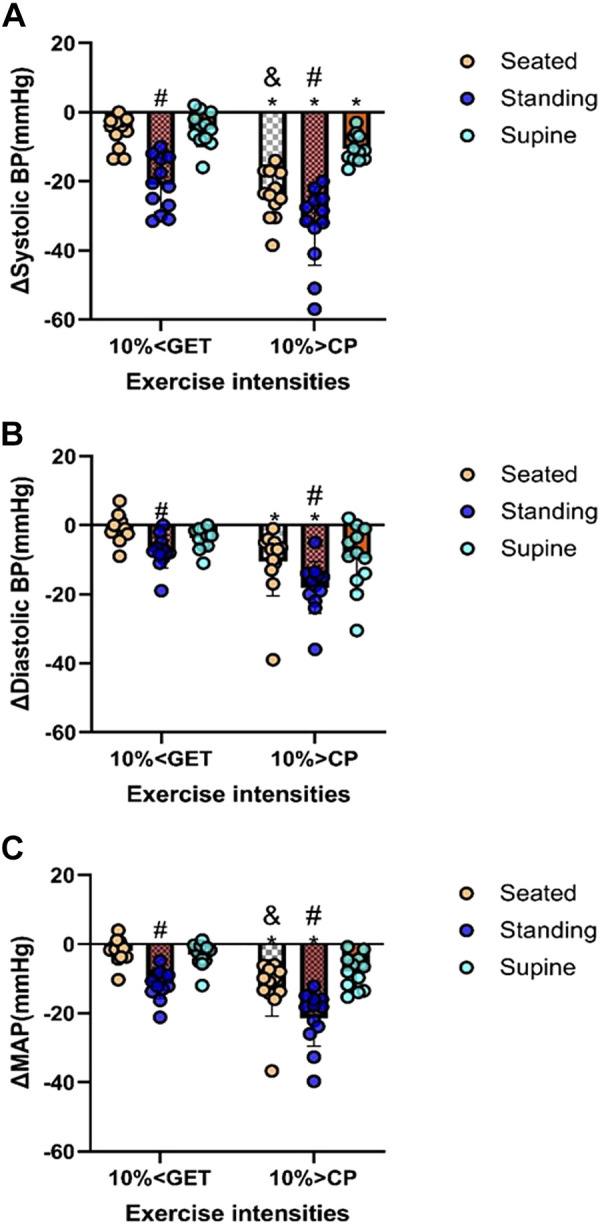
Combined effect of exercise intensities (10% > CP and 10% < GET) combined with different recovery postures (seated, supine, and standing) on the minimum reduction of systolic (*A*), diastolic (*B*), and mean arterial blood pressures (*C*) from baseline during the recovery. *Significant differences between 10% > CP and 10% <GET to the corresponding posture (*P* < 0.05); #standing significantly different from seated and supine posture within the same exercise intensity (*P* < 0.05); &significant differences between seated and supine at 10% > CP only (*P* < 0.05).

The group means ± SD responses of systolic, diastolic, and MAP from rest to the end of the recovery period for both intensities and in each posture are displayed as changes from the resting baseline value (i.e., Δ mmHg) in Fig. 1 and as absolute values (i.e., mmHg) in Fig. 2. Moreover, summary data indicating the greatest reduction in blood pressure during the recovery period at each intensity and within each posture are displayed in Fig. 3.

Within each exercise intensity, systolic, diastolic, and MAP were reduced to a greater extent during the recovery period in standing compared with the seated and supine recovery postures (all *P* < 0.05; Figs. 1, 2, and 3). When different exercise intensities were compared, changes in systolic, diastolic, and MAP during the recovery period were exacerbated following exercise performed at 10% > CP when compared with 10% < GET in standing and seated postures (all *P* < 0.05; Figs. 1, 2, and 3). However, exercise intensity-related differences in the magnitude of postexercise hypotension for diastolic blood pressure and MAP were not evident in the supine posture (all *P* > 0.05; Figs. 1, 2, and 3). Figures 1 and 2 show the specific time points during the recovery at which significant differences were observed.

### Body Temperature

**Figure 4.**
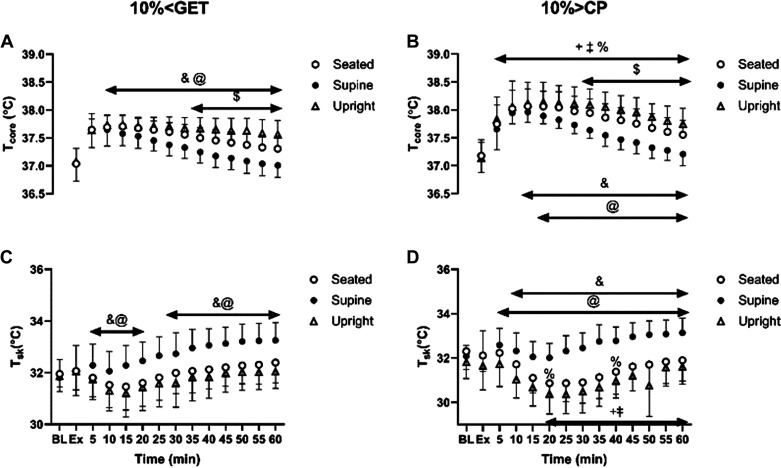
Effect of different exercise intensities [10% < GET (*A*, *C*) and 10% > CP (*B*, *D*)] combined with different postures (seated, supine, and standing) on core and skin temperatures response during exercise and throughout the 1-h recovery. &Significant differences between seated and supine within each intensity (*P* < 0.05); $significant differences between seated and standing within the same exercise intensity (*P* < 0.05); @significant differences between supine and standing within the same exercise intensity (*P* < 0.05); +10% > CP different from 10% < GET at seated posture (*P* < 0.05); ‡10% > CP different from 10% < GET at standing posture (*P* < 0.05); %significant differences between 10% > CP and 10% < GET at supine position (*P* < 0.05).

The group means ± SD skin and core temperatures at rest, immediately postexercise and throughout 1 h of recovery within each condition are displayed in Fig. 4. At both intensities, T_core_ in supine posture was lower than seated and standing postures from 10 (10% < GET) and 15 (10% > CP) min of the recovery onward, whereas it was lower in the seated compared with the standing posture from 30 (10% > CP) and 35 (10% < GET) min onward (Fig. 4, *A* and *B*). When different exercise intensities within the same posture were compared, T_core_ was higher following exercise performed at 10% > CP compared with 10% < GET from 5 min of recovery onward.

At both exercise intensities, T_sk_ was greater during the recovery period in the supine posture when compared with the seated and standing postures (Fig. 4, *C* and *D*). When different exercise intensities within the seated and standing postures were compared, T_sk_ was higher in the recovery phase following exercise performed at 10% > GET when compared with 10% > CP. However, in the supine posture, these differences were less pronounced, with significant differences emerging only at the 20- and 40-min time points of recovery (Fig. 4, *C* and *D*).

### Vascular Compliance, Heart Rate, and Oxygen Pulse

**Figure 5.**
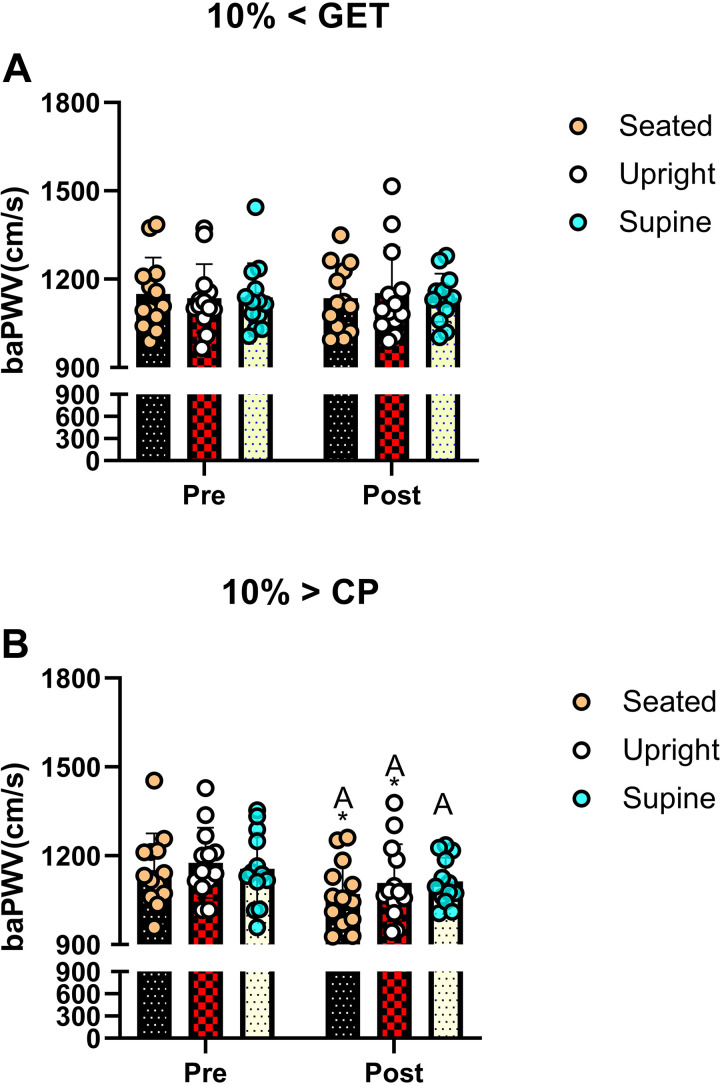
Effect of exercise intensities [10% < GET (*A*) and 10% > CP (*B*)] combined with different recovery posture on vascular compliance at baseline and following 1-h recovery. *Significant differences between 10% > CP and 10% < GET to the corresponding posture (*P* < 0.05); The letters A in *B* indicate significant differences between pre- and 1-h postexercise within the same posture (*P* < 0.05).

Postexercise, baPWV was reduced compared with baseline following 10% > CP in all postures; however, this was not the case following 10% < GET across all postures (Fig. 5, all *P* < 0.05). However, although baPWV was consistently reduced from baseline following exercise performed at 10% > CP (supine, seated, and standing), postexercise baPWV was lower within 10% > CP when compared with 10% < GET in the standing and seated postures only. Finally, baPWV was not different between different postures following 1-h recovery of the exercise (all *P* > 0.50).

**Figure 6.**
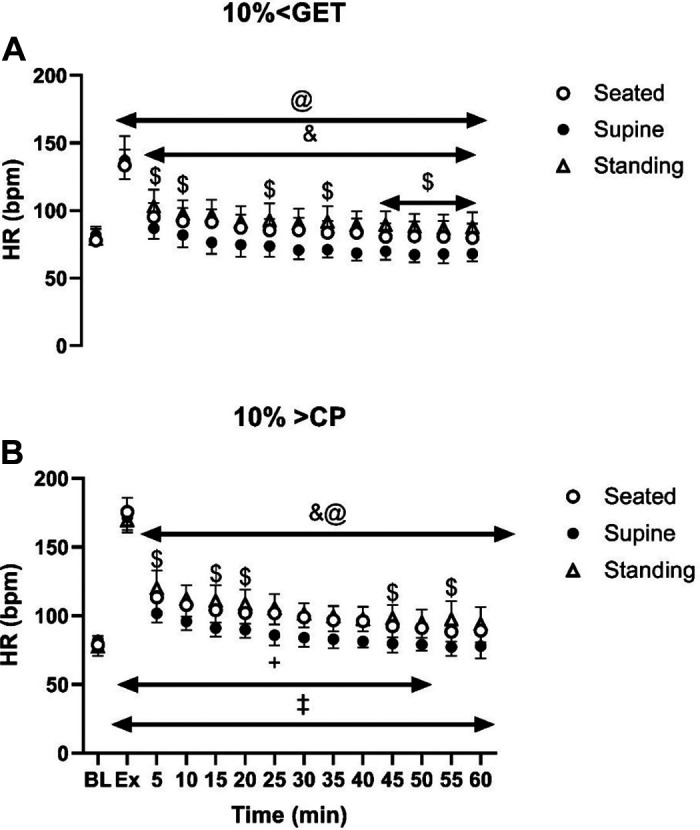
Effect of different exercise intensities [10% < GET (*A*) and 10% > CP (*B*)] combined with different postures (seated, supine, and standing) on heart rate (HR) response during exercise and throughout the 1-h recovery. &Significant differences between seated and supine within each intensity (*P* < 0.05); $significant differences between seated and standing within the same exercise intensity (*P* < 0.05); @significant differences between supine and standing within the same exercise intensity (*P* < 0.05); +10% > CP different from 10% < GET at seated posture (*P* < 0.05); ‡10% > CP different from 10% < GET at standing posture (*P* < 0.05); %significant differences between 10% > CP and 10% < GET at supine posture (*P* < 0.05).

Within each exercise intensity, HR was higher in the seated recovery posture than in the supine posture and was also greater following exercise at 10% > CP when compared with 10% < GET (Fig. 6).

Oxygen pulse during the recovery was not different between different exercise intensities (*P* = 0.36) or between different postures (*P* = 0.50). Furthermore, no interaction was found among exercise intensities, postures, and time points (*P* = 0.42).

### Hematological Variables

Postexercise hemoglobin concentration was greater in the seated compared with the supine posture, whereas at the end of the recovery, it was lowest in the seated compared with the supine and standing postures (all *P* < 0.05). Moreover, at both intensities, hematocrit in the seated recovery posture was lower than the supine posture at the end of the recovery (*P* < 0.05). The plasma volume expansion from postexercise to the end of the recovery was greater in supine than in seated and standing postures across both intensities (*P* < 0.05). Furthermore, plasma volume expansion from postexercise to the end of the recovery was greater in seated than in standing at the intensity above CP (*P* < 0.05) and was greater in the intensity above CP than GET at seated posture only (*P* < 0.05).

## DISCUSSION

This study reveals two major findings. First, PEH exhibited a greater magnitude following exercise intensity above critical power when recovery was undertaken in an upright posture (standing and seated) compared with following exercise performed below the GET within the same posture. Second, in the supine position, the magnitude of PEH was not influenced by exercise intensity for diastolic pressure and thus MAP, aligning with our study hypothesis. Collectively, the attenuation of intensity-based differences in the magnitude of PEH in the supine position suggests that such intensity-based differences are mediated, at least in part, by the effect of supra-CP exercise on venous pooling when recovery is performed in standing or seated posture.

We observed that when high-intensity exercise (10% > CP) was coupled with postural-induced orthostatic stress, the magnitude of PEH surpassed that of moderate exercise intensity within the same posture (Figs. 1, 2, and 3). A plausible physiological mechanism could be an increased vascular compliance (23), leading to greater venous pooling, resulting in a more pronounced PEH. Our results support this hypothesis, as baPWV was lower in 10% > CP in both seated and standing postures compared with 10% < GET in the same postures (see Fig. 5) but not in the supine position. Venous return, central blood volume, left ventricular end-diastolic volume, and stroke volume have been demonstrated to be augmented in the supine versus upright body positions, owing to volume shifts related to the loss of the impact of gravity and the hydrostatic column (24–26). Such alterations can influence cardiopulmonary and arterial baroreceptors, which in turn have the potential to influence the sympathetically mediated alterations in vascular conductance during exercise and the postexercise recovery period (19, 27–29). Hence, it is likely that the increased magnitude of PEH following exercise performed above critical power observed in our previous study (9) was related to a reduction in venous return secondary to increased postexercise venous pooling. This interpretation is supported by other findings whereby PEH was completely nullified via maintenance of the muscle pump during the recovery period (29). The integrative signaling mechanisms that mediate the increased vascular conductance and venous pooling following exercise performed above critical power are unknown particularly in regard to how much is mediated through humoral versus autonomic control.

The present findings align with our prior study indicating that critical power is a key threshold that determines the magnitude of PEH (9). However, both studies contrast with the finding of Scott et al. (11), where higher exercise intensity combined with moderate orthostatic stress (–40 mmHg) via lower body negative pressure did not augment PEH compared with exercise performed below the gas exchange threshold. This discrepancy could be attributed to the greater effect of baroreceptor unloading in a standing posture compared with the moderate orthostatic stress induced by lower body negative pressure (13). Another methodological difference is that exercise performed above critical power in this and our previous work (9) was performed to task failure, whereas this was not the case in the study of Scott et al. (11), and the attainment of task failure may be important for production of vasodilatory factors, both locally and humorally. Hence, as suggested in our previous work (9), the greater magnitude of PEH seen following exercise performed above critical power could be unique to the magnitude of nonoxidative metabolism, rather than the effect of exercise intensity per se. Further studies are required to test this hypothesis.

The observed increase in vascular compliance following the 10% > CP trial, leading to lower baPWV compared with 10% < GET, could be partly attributed to a greater postexercise hyperthermia, resulting in greater postexercise vasodilation (18). Our results support this supposition, as postexercise core temperature was higher in 10% > CP compared with 10% < GET despite matched work volume (see Fig. 4). In addition, as shown by Piepoli et al. (30), PEH persists following maximal exercise despite enhanced sympathetic tone and reduced vagal tone, largely due to persistent vasodilation. Finally, a sustained postexercise vasodilation could reflect histamine receptor activation and withdrawal of sympathetic nerve activity following the higher-intensity exercise, which would be evident in standing and seated postures (31, 32).

## LIMITATIONS

Although we successfully addressed the combined stressors’ effect of exercise intensity and postural-induced PEH in a healthy Asian male population, three major limitations necessitate further investigation. First, the lack of female participants in this study prompts future research to explore potential sex differences in PEH under varying levels of orthostatic stress induced by different postures and exercise intensities. However, considering that previous studies indicate no significant sex differences in the magnitude of PEH (33, 34), similar observations may be anticipated. Second, the lack of direct assessment of muscle sympathetic nerve activity in this study precludes an examination of whether the greater magnitude of PEH following the 10% > CP trial is mediated at least in part from attenuated baroreceptor sensitivity relative to local factors, including adrenoreceptor sensitivity. Third, we did not examine the stroke volume and cardiac output to ascertain their role in blood pressure maintenance. Finally, the validity of assessment of changes in blood volume is potentially affected by the introduction of red cells to the circulation from the spleen, or alterations in the venous-versus-whole blood distribution, which could impact pressure by way of volume. For example, stronger splenic constriction at the higher work rate would lead to an overestimation of the reduction in plasma volume, perhaps by ∼1%, and thereby seem unlikely to account for the PEH or differences between conditions.

### Implications of This Study

Although the repeated occurrence of PEH is acknowledged to be a key physiological mechanism to lower resting blood pressure in healthy and diseased populations (35), PEH is a double-edged sword, potentially leading to postexercise syncope following high-intensity exercise in a standing posture, with almost 50% of the population developing presyncopal symptoms (10). This study supports this hypothesis, as we observed a greater magnitude of PEH following high-intensity exercise in a standing posture compared with seated or supine postures. Furthermore, the results suggest that changing posture to supine or seated could effectively mitigate the magnitude of PEH, a consideration when muscle pump availability is limited, such as following volitional exhaustion from exercise. Moreover, our findings shed light on the mechanisms underpinning exercise intensity-linked differences in the magnitude of PEH following exercise.

### Perspectives and Significance

Exercise performed above critical power followed by recovery in a standing or seated posture resulted in a greater magnitude of PEH when compared with exercise performed below GET. However, this intensity-based difference in the magnitude of PEH was not apparent when recovery was in the supine posture.

## DATA AVAILABILITY

All of the data will be available upon request to the authors.

## GRANTS

This study was supported by Hubei Normal University Research Grant HS2021RC012 (to T.-H. L).

## DISCLOSURES

No conflicts of interest, financial or otherwise, are declared by the authors.

Zachary Schlader is an editor of *American Journal of Physiology-Regulatory, Integrative and Comparative Physiology* and was not involved and did not have access to information regarding the peer-review process or final disposition of this article. An alternate editor oversaw the peer-review and decision-making process for this article.

## AUTHOR CONTRIBUTIONS

R.P.G., I.-L.W., Z.L., H.-Y.L., H.W., N.K., T.-H.L., and F.W. conceived and designed research; T.-H.L. performed experiments; T.M., Z.J.S., J.D.C., S.K., N.F., T.-H.L., and F.W. analyzed data; R.P.G., Z.J.S., J.D.C., S.K., N.F., T.-H.L., and F.W. interpreted results of experiments; X.L. and T.-H.L. prepared figures; T.M., Z.J.S., H.-Y.L., and T.-H.L. drafted manuscript; R.P.G., T.M., Z.J.S., J.D.C., S.K., N.F., I.-L.W., Z.L., H.-Y.L., H.W., H.Z., C.-Y.G., T.-H.L., and F.W. edited and revised manuscript; X.L., R.P.G., T.M., Z.J.S., J.D.C., S.K., N.F., I.-L.W., Z.L., H.-Y.L., H.W., H.Z., N.K., C.-Y.G., T.-H.L., and F.W. approved final version of manuscript.
